# Medicine Use in Chronic Diseases

**DOI:** 10.3390/pharmacy11030100

**Published:** 2023-06-12

**Authors:** Elizabeth Unni

**Affiliations:** Social, Behavioral, and Administrative Sciences, Touro College of Pharmacy, New York, NY 10027, USA; elizabeth.unni@touro.edu

Welcome to this Special Issue on “Medicine Use in Chronic Disease” in *Pharmacy*, an open-access journal focusing on pharmacy education and practice. In 2022, a special call was issued to researchers to share their work on the use of medicine in chronic diseases in the form of articles, reviews, meta-analyses, and commentaries. According to the Centers for Disease Control and Prevention, a chronic disease is a condition that lasts a year or more and necessitates ongoing medical attention, limits daily activities, or both. Approximately one in every three adults worldwide suffers from multiple chronic conditions.

Individuals diagnosed with chronic conditions require optimal drug therapy to achieve the best clinical outcomes and health-related quality of life. Most, if not all, of these individuals must take medications for the rest of their lives and must develop a therapeutic alliance with their healthcare providers. Steps must be taken by providers to ensure that their patients have a safe, effective, and compatible medication use experience. Medicine use in chronic diseases can be defined in a variety of ways, including the individual’s experience with taking medications, healthcare providers’ perspectives on how medicines are used by patients, the impact of quality, access, and cost on medicine use, patterns of medicine use, the impact of medicine use on healthcare utilization, factors that influence medication use, challenges in medication use, interventions that promote medication use, and methods to measure medicine use. The goal of this Special Issue is to gather studies that examine the medication use experiences of individuals with chronic diseases.

A significant part of medication use in individuals with chronic conditions is their engagement in their care and their ability to be a part of the treatment decision-making process. Walker et al. studied the need for patient input while choosing the medication formulation for acute agitation in behavioral emergencies [1]. Understanding these preferences and prescribing accordingly is critical to improving the acceptability of the medicine, medication adherence, and therapeutic alliance. Engaging patients in their care involves patient education and communicating with patients about the benefits and risks of medicines. Aldarwesh et al., in a study among patients with autoimmune rheumatic disease where hydroxychloroquine has the potential for ocular toxicity, reported a lack of communication between patients and providers about the risk for retinopathy [2]. On the other hand, a case study reported by Undeberg et al. from a rural town in the United States showed the importance of respecting the patient’s decision even when it does not align with the recommendation from the provider [3]. 

Medication adherence, or taking the medication as prescribed by the provider, is an integral part of optimal clinical outcomes and is heavily reliant on the medication use experience of individuals. In their paper, Unni et al. developed a new hierarchical model to explain medication adherence, with health literacy as the basis, and illness beliefs, medication beliefs, and self-efficacy as hierarchical steps to achieve medication adherence [4]. Makhinova and colleagues examined the medication use experience of patients with persistent asthma and the potential influence of pharmacists in improving medication adherence [5]. The study showed how pharmacists were able to reduce the treatment belief barriers of patients, which impacts their medication adherence. Shiyanbola et al. published a randomized control trial protocol that compared an established adherence intervention tool for type 2 diabetes known as Healthy Living With Diabetes and a new tool developed by the authors [6]. The new tool, named PEERS-EXCEL, was developed as a culturally competent intervention tool for African Americans. The authors hypothesized that there would be a significant improvement in medication adherence and HbA1C levels among African Americans when using a culturally adapted intervention tool. The same research team also had another study where they showed how the medication use experience of African American patients living with type 2 diabetes can be improved by engaging community dwelling African American patients as part of the intervention development team [7]. The authors were able to further refine and implement their culturally competent intervention based on feedback from these community stakeholders. Ali et al. described the personal, financial, and structural barriers that either disable or delay individuals in accessing medicines for type 2 diabetes, especially those who visit the Federally Qualified Health Centers [8]. Based on their study, interventions that target personal barriers, such as poverty, health status, and psychological distress; financial barriers, such as out-of-pocket costs and employment; and structural barriers, such as health center funding programs for medicine access, should be targeted to achieve improved adherence to medicines.

Adhering to treatment guidelines and understanding the real-world evidence (RWE), especially for new treatments, are needed to improve the medication use experience of patients. Migotsky et al. undertook a literature review to understand the real-world evidence of the new drugs used in the treatment of sickle cell disease [9]. Though the review showed that there were studies that determined the efficacy of these new drugs, there was a paucity of patient-reported outcomes and health-related quality of life studies. Having more RWE studies that show the actual medicine use experience of patients can be of significant importance in chronic disease conditions. In their study, Almodovar et al. compared type 1 diabetes patients on insulin alone and on insulin and adjuvant therapies [10]. They found that there was no significant change or decrease in HbA1C levels in patients over a 7-year period. The study shows the importance of real-world evidence studies in understanding the medication use experiences of patients when they are not strictly monitored for adherence and access to medicines as in clinical trials. In fact, what improved medication adherence was older age, education, higher income, and the use of continuous glucose monitors. 

When prudent, treatment guidelines need to be followed, Brust-Sisti et al. argue for improving patient outcomes by increasing patient accessibility to newer treatments approved by the treatment guidelines, such as SGLT2 inhibitors for patients with heart failure with preserved ejection fraction. Despite the recommendation from the FDA and other treatment guidelines, prescriptions for SGLT2 inhibitors are still lacking [11]. Prudentio et al. from a rural primary care clinic in East Hawaaii demonstrated how having a pharmacist on the interdisciplinary care team can increase the uptake of new treatments such as SGLT2 inhibitors and GLP1 in patients with type 2 diabetes and cardiovascular diseases [12]. Using comprehensive medication management and provider education, pharmacists were able to optimize treatment for these patients and achieve optimal clinical outcomes. On the other hand, Schroeder et al., in their observational study of patients with acute myocardial infarction, examined whether deviation from the guidelines is always a bad case [13]. Their study findings refuted this claim and reported that there was a trade-off between the beneficial and detrimental effects of the guideline-based treatment in their study sample. They argue that at times, to improve the medication use experience of patients, the treatment guidelines may not be followed by the physicians.
